# Diagnosis of bacterial meningitis in Ghana: Polymerase chain reaction versus latex agglutination methods

**DOI:** 10.1371/journal.pone.0210812

**Published:** 2019-01-17

**Authors:** Nafiu Amidu, Benedict Boateng Antuamwine, Otchere Addai-Mensah, Abass Abdul-Karim, Azure Stebleson, Braimah Baba Abubakari, John Abenyeri, Afia Serwaa Opoku, John Eyulaku Nkukah, Ali Sidi Najibullah

**Affiliations:** 1 Department of Biomedical Laboratory Sciences, School of Allied Health Sciences, University for Development Studies, Tamale, Ghana; 2 Department of Medical Laboratory Technology, School of Allied Health Sciences, Kwame Nkrumah University of Science and Technology, Kumasi, Ghana; 3 Public Health Reference Laboratory, Northern Region, Tamale, Ghana; 4 Northern Regional Health Directorate, Ghana Health Service, Tamale, Ghana; Universita degli Studi di Parma, ITALY

## Abstract

Bacterial meningitis is a public health crisis in the northern part of Ghana, where it contributes to very high mortality and morbidity rates. Early detection of the causative organism will lead to better management and effective treatment. Our aim was to evaluate the diagnostic accuracy of Pastorex and Wellcogen latex agglutination tests for the detection of bacterial meningitis in a resource-limited setting. CSF samples from 330 suspected meningitis patients within the northern zone of Ghana were analysed for bacterial agents at the zonal Public Health Reference Laboratory in Tamale using polymerase chain reaction (PCR) and two latex agglutination test kits; Pastorex and Wellcogen. The overall positivity rate of samples tested for bacterial meningitis was 46.4%. *Streptococcus pneumoniae* was the most common cause of bacterial meningitis within the sub-region, with positivity rate of 25.2%, 28.2% and 28.8% when diagnosed using Wellcogen, Pastorex and PCR respectively. The Pastorex method was 97.4% sensitive while the Wellcogen technique was 87.6% sensitive. Both techniques however produced the same specificity of 99.4%. Our study revealed that the Pastorex method has a better diagnostic value for bacterial meningitis than the Wellcogen method and should be the method of choice in the absence of PCR.

## Introduction

Bacterial meningitis is a huge public health problem especially in the northern parts of Ghana, making its contribution to mortality and morbidity rates difficult to overlook. Whilst the global outlook on meningitis indicates a mortality rate of 2–30%, that of Ghana is estimated to be between 36–50%, with majority of the cases occurring within the northern zone [1–3]. The location of northern Ghana within the imaginary boundaries of the meningitis belt of sub-Saharan Africa accounts for the high burden of the infection in the region [4, 5].

This life-threatening infection of the central nervous system is mostly caused by *Haemophilus influenzae* type B, *Neisseria meningitidis* and *Streptococcus pneumoniae*. Outbreaks of meningitis infection by these organisms have become increasingly associated with children and young adults in overcrowded homes and boarding schools [6]. *N*. *meningitidis*, the cause of meningococcal infection has been the leading cause of bacterial meningitis in West Africa, especially among infants and children [7–9]. Recent sporadic outbreaks of pneumococcal meningitis caused by *S*. *pneumoniae* in Ghana and other parts of West Africa among older children and adolescents is an issue of concern [10–13].

Interventional approaches such as large-scale vaccination, adopted to curb the spread of meningitis have resulted in a gradual decline in the frequency of the infection. This notwithstanding, periodic outbreaks of meningitis are not uncommon within the meningitis belt [14, 15]. In 2012, with the support of Gavi, the Vaccine Alliance, two meningitis vaccines were introduced in Ghana; the MenAfriVac and the PCV13. While the PCV13 was incorporated into the national infant immunization program using a three-dose schedule at 6, 10, and 14 weeks of age with no booster, the MenAfriVac was administered to young children and adults [15, 16]. However, despite high reported administrative coverage of the three doses of PCV13, outbreaks of pneumococcal meningitis continue to occur in the region. In 2015–2016 meningitis season, 886 patients were reported with suspected meningitis in the Brong-Ahafo region, with majority of cases occurring in young adults [10]. Additionally, in the most recent outbreak in 2016, the Ghana Health Service reported over 400 suspected cases of meningitis nationwide with 90 (22.5%) deaths and significant morbidities. Among the confirmed cases, *S*. *pneumoniae* was identified as the leading causative organism, although *N*. *meningitidis* (strain NM W) and *N*. *meningitidis* type C were also isolated [17].

Inaccurate diagnosis, together with overcrowding, coughing and sneezing, sharing of belongings, exposure to second hand smoke and poor sanitation among others, have been linked to high risk pathogen carriage and transmission [6, 18]. Early detection of the causative organism will lead to better management and effective treatment, ensuring proper containment of the spread of the infection.

The gold standards for the diagnosis of bacterial meningitis remain the classic culture and the PCR methods [19]. In sub-Saharan countries including Ghana, neither of these two diagnostic methods is available outside the capital, large cities and reference laboratories [20]. It is therefore not unusual to find the old non-culture test method (leukocyte cell count and Gram stain microscopy) widely employed for the diagnosis of bacterial meningitis in the District laboratories. Although rapid and cheap, the technique is limited in its identification of serogroups [21, 22], which makes it unreliable in the event of an outbreak as it is unable to guide the choice of a vaccine. The introduction of latex agglutination tests (LATs) for the identification of bacterial antigens in cerebrospinal fluid (CSF) of patients is gradually replacing the leukocyte cell count and Gram stain technique at the District laboratories [23, 24]. The LATs employ antigen-specific antibodies on latex particles to detect bacterial antigens. CSF sample suspected to contain a target bacteria species will form a visible agglutination when mixed with reagents containing the latex particles. This method can detect antigens of 5 pathogens in CSF including; *Escherichia coli* K1 antigen, *N*. *meningitidis* ABCY or W135 antigens, *S*. *pneumoniae* antigen, Group B streptococcus antigen and *H*. *influenzae* type B antigen [25]. Pastorex and Wellcogen diagnostic test kits have become increasingly available in our District laboratories for the detection of bacterial serogroups that cause meningitis [22, 26]. However, information on the sensitivity and specificity of these rapid diagnostic test kits are lacking within our setting. Such information would help adopt better strategies in the management of meningitis outbreaks which often start from the rural areas.

The study was therefore aimed at evaluating the diagnostic accuracy of Pastorex and Wellcogen test kits in the detection of bacterial antigens in meningitis, for use in rural areas and/or at the patient’s bedside.

## Materials and methods

### Study setting and design

The Brong-Ahafo, Northern, Upper East and Upper West Regions make up the northern zone of Ghana, which lie within the meningitis belt. The zone with a population of about 6.8 million, in its 77 districts accounts for about 59.1% of the total land area of Ghana. The 3 northern regions have similar rainfall patterns, starting from May to September while that of the Brong-Ahafo region spans from April to October. Brong-Ahafo region also has a dry season running from November to March while that of the 3 northern regions runs from October to April. The evaluation was conducted at the Public Health Reference Laboratory which is centrally located in Tamale within the northern zone. CSF specimen from 41 Districts within the zone were collected from suspected cases of acute bacterial meningitis from October 2016 to January 2017 using WHO case definitions [27]. The CSF samples were anonymized prior to access and analysis.

### Patients

All patients with suspected cases of meningitis presenting at mission, district and regional hospitals within the northern zone received a lumbar puncture. Suspected case of meningitis was defined as sudden onset of fever (>38.5 °C) and a combination of any of the following clinical symptoms: reduced level of consciousness, stiff neck, bulging fontanels, fit(s) if aged between 6 months and 5 years or partial seizures [28]. The CSF specimens were stored in Trans-isolate media and transported to the Tamale reference laboratory on ice packs where they were stored at -20°C prior to testing. The patients were treated with ceftriaxone or ampicillin.

### Laboratory method

CSF samples which were anonymized were tested for bacterial antigen employing two latex agglutination techniques; Pastorex and Wellcogen test kits. Each can detect antigens of 5 organisms; *E*. *coli* K1 antigen, *N*. *meningitidis* ABCY or W 135 antigens, *S*. *pneumoniae* antigen, Group B *Streptococcus* (GBS) and *H*. *influenzae* type B (Hib) antigen.

#### Pastorex latex agglutination test

The Pastorex kit (Bio-Rad, Marnes-la-Coquete, France) included four suspensions of latex particles, three for the individual detection of *N*. *meningitidis* serogroups A, C, and B/*E*. *coli* K1 individually and one for combined Y/W135 capsular polysaccharide antigen detection. The Pastorex kits were used according to the manufacturer’s recommendations. Briefly, the CSF specimens were centrifuged for 5 min at 3000 g to obtain the supernatant, which was then heated in a dry incubator at 100°C for 3 min. The disposable card was gently rocked for 3 min after 40 μL of the supernatant had been placed on the specific locations with their corresponding reagent. The appearance of agglutination within 10 min was observed with the naked eye.

#### Wellcogen latex agglutination test

The components of the Wellcogen bacterial antigen kit (Abbot-Murex, Illinois, US) included one suspension of latex particles with a combination of adsorbed antibodies for the detection of *N*. *meningitidis* combined serogroups A, C, Y, W135 and another latex suspension with *N*. *meningitidis* serogroup B/Escherichia coli K1 antibodies. The Wellcogen kits were used according to the manufacturer’s recommendations. Briefly, the CSF samples were heated for 5 minutes at 100°C in a water bath, cooled to room temperature and then centrifuged for 5 min at 3000 g. The disposable card was gently rocked for 3 min after 40 μL of the supernatant had been placed on the specific locations with their corresponding reagent. The appearance of agglutination within 10 min was observed with the naked eye.

#### Real time PCR and serotyping of pathogens

As it was not possible to obtain rapidly a sufficient number of culture-positive CSF, PCR testing was used as the gold standard for assessing latex agglutination results [7, 19]. The laboratory technicians who performed the latex agglutination tests were blinded to the results of the PCR assay. Species-specific quantitative PCR (qPCR) assays for detection of pneumococcus, meningococcus and *H*. *influenzae* were conducted using the autolysin gene (lytA), the CU, Zn superoxide dismutase gene (sodC) and the protein D encoding gene (hpd) respectively as previously described [29, 30]. RNaseP gene assay was performed on all CSF specimens to confirm samples of human origin and the integrity of the CSF specimens. Positivity for each of the targets was deduced using cycle threshold (CT) values. CTs of ≤36 were considered as positive.

#### Serogroup and serotype specific qPCR assays

Meningococcal serogrouping and *H*. *influenzae* serotyping were performed by direct qPCR as previously described [30]. Targets for the mentioned pathogens included sacB, synD, synE, synG, xcbB, synF genes for serogroups A, B, C, W, X, Y respectively. For *H*. *influenzae*, the following serotypes were screened: acB (Hia), bcsB (Hib), ccsD (Hic), dscE (Hid), ecsH (Hie) and bexD (Hif).

### Statistical analysis

Demographic and clinical data of suspected cases were collected with designed case report forms and entered into Microsoft Excel. Data analysis was carried out using Graph-Pad Prism version 6 and the Medical Statistical tool. Continuous data are presented as mean and standard deviation while categorical data are presented as frequency and percentages. The diagnostic values of the two techniques were analysed for sensitivity, specificity, positive and negative likelihood ratios, per the standard statistical methods.

### Ethical consideration

Specimens were collected for analyses in accordance with standard practice as required during the routine clinical management of patients [28]. Consequently, informed consent was not sought and the study was not reviewed by an ethical committee. However, permission was obtained from the Ghana Health Service Regional Directorate to use the data on meningitis from the Public Health Reference Laboratory of the region for the purpose of academic publications.

## Results

### General characteristics of tested CSF specimen according to age and gender of patients

The study was conducted on 330 CSF samples obtained from patients with suspected cases of meningitis across 41 Districts in the northern zone of Ghana. There was fairly equal distribution of the cases of meningitis between gender; 53.0% and 47.0% for males and females respectively with an overall mean age of 23.7±19.8 as shown in Table 1.

**Table 1:** The distribution of suspected cases of bacterial meningitis by age groups and gender.

Age (yrs.)	Total (330)	Males (175)	Females (155)	P-value
Overall	23.7±19.8	23.1±20.4	24.4±19.0	0.5851
<5	33(10.0%)	20(6.1%)	13(3.9%)	0.4626
5–18	149(45.2%)	80(24.2%)	69(20.9%)	0.9118
>18	148(44.8%)	75(22.7%)	73(22.1%)	0.5059

### General comparison of the diagnostic value of the LATs with PCR

Our study explored the distribution of positive and negative meningitis samples that were detected by each of the latex agglutination methods in comparison with that of the PCR. Both latex agglutination techniques detected a little fewer meningitis positive samples compared to the gold standard technique (PCR). Positive meningitis cases detected by the Pastorex latex agglutination method were more comparable to that of the PCR than the Wellcogen technique (Table 2). Additionally, more negative meningitis samples were recorded with Pastorex (54.6%) and Wellcogen (59.1%) techniques compared to the PCR (53.6%) as shown in Table 2.

**Table 2:** Diagnosis of bacterial meningitis in 330 CSF samples using latex agglutination methods and PCR.

Results	Pastorex	Wellcogen	PCR
**Positive**	150(45.5%)	135(40.4%)	153(46.4%)
**Negative**	180(54.6%)	195(59.1%)	177(53.6%)

True positivity/negativity of the Pastorex and Wellcogen techniques for detection of bacterial antigens in CSF were determined by comparing the LATs results with that of the PCR. Truly positive results for detection of bacterial antigens by the Pastorex method was observed to be 97.4% and 87.6% for the Wellcogen method as shown in Table 3. This raises concerns about the sensitivity of the Wellcogen technique. Truly negative results for detection of bacterial antigens by the Pastorex and Wellcogen methods were not different, as both were observed to be 99.4% (Table 3).

**Table 3:** Cross tabulation of latex agglutination method versus PCR for detection of bacterial meningitis.

Latex Agglutination Method		PCR	
**Pastorex**		**Positive (153)**	**Negative (177)**
	**Positive (150)**	149(97.4%)	1(0.6%)
		**TP**	**FP**
	**Negative (180)**	4(2.6%)	176(99.4%)
		**FN**	**TN**
**Wellcogen**		**Positive (153)**	**Negative (177)**
	**Positive (135)**	134(87.6%)	1(0.6%)
		**TP**	**FP**
	**Negative (195)**	19(12.4%)	176(99.4%)
		**FN**	**TN**

### The distribution of pathogens detected by the test method

In order to determine variations among the test methods in the detection of meningitis-causing pathogens, we assessed the number of pathogens detected by each test method. In all, four pathogens; *S*. *pneumoniae*, *N*. *meningitidis*, *H*. *influenzae* and *Streptococcus B* were detected. *S*. *pneumoniae* was shown to be the commonest cause of meningitis within the sub-region. The positivity rate rates were 28.2%, 25.2% and 28.8% for Pastorex, Wellcogen and PCR respectively as shown in Table 4. The Pastorex latex agglutination method for meningitis detection produced a similar result to that of the PCR (Table 4). On the other hand, the Wellcogen latex agglutination method was the only technique that detected *Streptococcus B* as a causative organism for meningitis.

**Table 4:** The Distribution of CSF samples according to pathogen detected, age group and test method.

Pathogens	Age groups (yrs.)	Pastorex	Wellcogen	PCR
***S*. *pneumoniae***	<5	4(1.2%)	3(0.9%)	5(1.5%)
	5–18	42(12.7%)	33(10.0%)	42(12.7%)
	>18	47(14.2%)	47(14.2%)	48(14.6%)
	**Total**	**93(28.2%)**	**83(25.2%)**	**95(28.9%)**
***N*. *meningitidis***	<5	3(0.9%)	1(0.3%)	3(0.9%)
	5–18	38(11.5%)	34(10.3%)	39(11.8%)
	>18	13(3.9%)	11(3.3%)	13(3.9%)
	**Total**	**54(16.4%)**	**46(13.9%)**	**55(16.7%)**
***H*. *influenzae***	<5	1(0.3%)	1(0.3%)	1(0.3%)
	5–18	1(0.3%)	1(0.3%)	1(0.3%)
	>18	1(0.3%)	1(0.3%)	1(0.3%)
	**Total**	**3(0.9%)**	**3(0.9%)**	**3(0.9%)**
***Streptococcus B***	<5	0(0.0%)	0(0.0%)	0(0.0%)
	5–18	0(0.0%)	3(0.9%)	0(0.0%)
	>18	0(0.0%)	0(0.0%)	0(0.0%)
	**Total**	**0(0.0%)**	**3(0.9%)**	**0(0.0%)**

### ROC curve and areas

**Fig 1:**
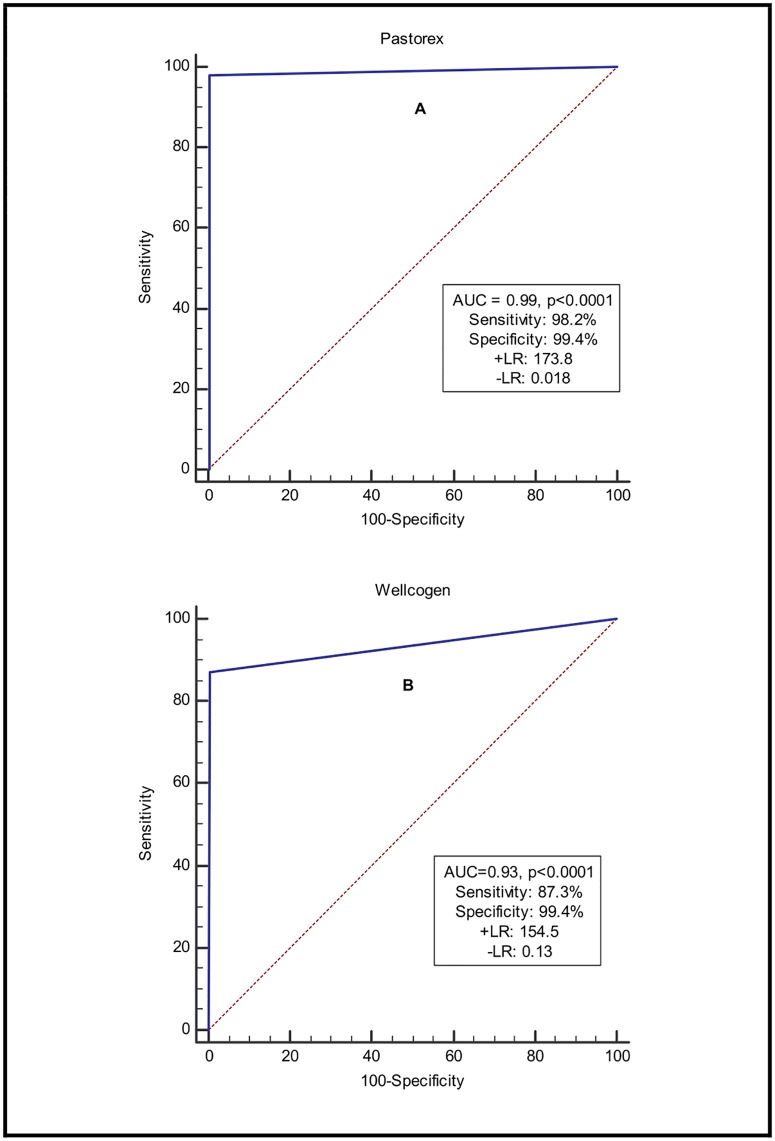
ROC analysis of latex agglutination methods and PCR in the detection of meningitis. AUC; area under the curve, +LR; positive likelihood ratio, -LR; negative likelihood ratio.

To further discriminate between the Pastorex and Wellcogen methods in the detection of meningitis causing bacteria, the receiver operating characteristics (ROC) curve analysis was performed. The ROC curve for the Pastorex technique showed a larger area under the curve (0.99, p<0.0001) and an increased sensitivity (98.2%) compared to the Wellcogen technique; (0.94, p<0.0001) and (87.3%) respectively as shown in Fig 1. This indicates that the Pastorex technique is a better diagnostic tool for bacterial meningitis than the Wellcogen technique. Additionally, higher positive likelihood ratio (+ LR) of 173.8 and a lower negative likelihood ratio (-LR) of 0.018 were obtained for the Pastorex latex agglutination method compared to the Wellcogen technique; 154.5 and 0.13 respectively as shown in Fig 1. This supports the conclusion that the Pastorex method offers some increasing discriminating ability in diagnosing true positive meningitis cases over the newly introduced latex agglutination meningitis test, Wellcogen.

## Discussion

Bacterial meningitis is a life threatening contagious disease, endemic in most developing countries, and thus requires early diagnosis [6, 31]. Worldwide, about 500,000 cases of meningococcal disease occur annually with 10% mortality rate [32]. The severity of these infections and the outbreaks they cause have necessitated the development and use of several rapid test kits for the diagnosis of bacterial meningitis. Concerns have however been raised about the diagnostic accuracy of these rapid test kits [21, 22].

The present study compared the diagnostic value of two kits; Pastorex latex agglutination test kit and Wellcogen latex agglutination test kit, employing PCR as the reference technique for the diagnosis of meningitis. The high positivity rate of meningitis among the young adult population demonstrates the changing epidemiology of bacterial meningitis as reported by Lexau and Brouwer [33, 34]. Though children and the aged are at a higher risk of developing meningitis [35–38], our findings of an increased positivity rate among the young adult population could be due to the collection of suspected CSF samples from adult populated sites that are prone to meningitis outbreaks as has been reported [39].

Additionally, the present study recorded a high positivity rate of pneumococcal meningitis contrary to reports from several studies[40–42] indicating a rather high incidence of meningococcal meningitis in the northern zone of Ghana. There may be a gradual shift from the dominance of meningococcus to pneumococcus in the aetiology of meningitis within the northern belt. New strains of *Streptococcus* species or increasingly dominant strains are emerging. A continual increase in pneumococcal meningitis was observed from 2000–2003 in the Kasena Nankana District of Northern Ghana [43].

The Latex agglutination test kits for the detection of bacterial meningitis is a simple and technically easy to perform method, requiring no expensive equipment whiles producing results in 10 minutes [44]. After comparing the Pastorex and the Wellcogen latex agglutination techniques with the PCR using different forms of analyses, closer similarities between the results of the Pastorex technique and the gold standard PCR were observed compared to the results of the Wellcogen technique. Cross tabulation of the results of the latex agglutination methods revealed a higher sensitivity for the Pastorex method compare to the Wellcogen technique, but the same specificity for both methods. The Wellcogen latex agglutination technique was less sensitive, despite the Wellcogen test method being a newer and improved test method over other latex agglutination techniques. It was therefore much anticipated that the Wellcogen test kit will have an increased sensitivity and specificity over other latex agglutination test methods including the Pastorex test kit, which have been demonstrated in earlier studies to be associated with poor diagnosis of meningitis [28, 45, 46].

In our further quest to discriminate between the two techniques, so as to adopt a more reliable rapid latex agglutination technique, the ROC analysis was performed. Despite the Wellcogen method showing similar diagnostic specificities with the Pastorex, its sensitivity was far less than that of the Pastorex technique. A much larger area under the curve and a positive likelihood ratio above 10 [47] for the Pastorex technique supports our arguments that the Pastorex method is a better diagnostic method than the Wellcogen technique.

## Conclusion

Our study has shown that the Pastorex method has a better diagnostic value for bacterial meningitis than the Wellcogen method. A diagnostic sensitivity of 97.4% and a specificity of 99.4% in the detection of bacterial meningitis by the Pastorex technique are remarkable. The study also found a high positivity rate of bacterial meningitis within the northern zone of Ghana to be 46.4%, with *S*. *pneumoniae* being the most common causative organism in the sub-region.

## Supporting information

S1 Dataset (ODS)
